# The emerging dental workforce: long-term career expectations and influences. A quantitative study of final year dental students' views on their long-term career from one London Dental School

**DOI:** 10.1186/1472-6831-9-35

**Published:** 2009-12-23

**Authors:** Jennifer E Gallagher, Resmi Patel, Nairn HF Wilson

**Affiliations:** 1 Oral Health Services Research & Dental Public Health, King's College London Dental Institute, At Guy's, King's College and St Thomas' Hospitals, London, UK; 2 Formerly Oral Health Services Research & Dental Public Health, King's College London Dental Institute, King's College London Dental Institute At Guy's, King's College and St Thomas' Hospitals, London, UK; 3 Office of the Dean and Head of School, King's College London Dental Institute At Guy's, King's College and St Thomas' Hospitals, London, UK

## Abstract

**Background:**

Research into the motivation and expectations of the emerging workforce and their short-term expectations has already been reported with a view to informing professional and policy decisions. The objective of this component of the research programme was to examine the long-term goals and perceived influences on final year dental students' professional careers.

**Methods:**

Univariate analysis of a self completed questionnaire survey of all final year dental students from King's College London, comprising questions on demography, long-term career goals and influences, proposed commitment to dentistry, commitment to healthcare systems and the influences thereon. Statistical analysis included Chi Squared tests for linear association.

**Results:**

Ninety per cent of students responded to this survey (n = 126), the majority of whom were aged 23 years (59%), female (58%) and Asian (70%). Long-term career goals were fairly evenly split between 'dentist with a special interest' (27%), 'primary dental care practitioner' (26%) and 'specialist' (25%), with 19% not certain. Only 60% of total respondents anticipated working full-time in the long-term (79% males cf 52% females; p = 0.00). The vast majority of respondents (≥80%) identified 'work-life balance', 'financial stability' and 'professional development' as 'important' or 'very important' influences on the number of future sessions. Females were significantly more likely to rate childcare commitments as an important influence on their future working capacity compared with males (p = 0.00). A wide range of factors were considered important or very important in making the NHS attractive, led by support for professional development (88%) and feeling valued by patients (88%), as well as funding, time with patients, rewards for prevention and practical issues such as dental materials and premises. Females were significantly more likely than males to be attracted to work within the NHS by 'childcare support' (p = 0.02), 'retraining facilities after career break' (p = 0.01), 'assistance with student debt' (p = 0.01) and 'incentives to work in deprived areas'.

**Conclusion:**

Long-term career plans of new graduates from this London Dental School commonly embrace opportunities for professional development as well as personal issues such as work/life balance and financial income. Significant differences were identified between male and females long-term plans and influences. The implications of these findings are discussed.

## Background

### Introduction

The dental workforce is an important healthcare resource [1]. It is therefore important to understand the views and career expectations of new entrants to the dental profession to protect and develop this resource. This paper is the fourth and final paper in a series reporting a dual methodological study to look at the motivation and professional career expectations of final year dental students at King's College London Dental Institute [KCLDI]. The development of the questionnaire instrument was informed by the literature and preliminary qualitative research. The motivation for choosing a career in dentistry has been reported, highlighting students' choice of dentistry as a 'contained professional career in healthcare' [2], together with the importance of a range of factors, including 'features of the professional job' and 'healthcare/people' [3]. The motivation for actively choosing dentistry as a professional career in healthcare was supported by concurrent research conducted with Vocational Dental Practitioners [VDPs] across England and Wales [4], as a 'financially lucrative, contained career in healthcare, with professional status, job security and the opportunity to work flexibly'. Short-term career aspirations of students involve 'achieving professional status within a social context', with a mix of personal, social, professional and financial goals. Location of future practice in the short-term was significantly associated with ethnicity [5], with Asian students significantly more likely to identify 'proximity to family' and 'being in an 'urban area', than their white counterparts, as important influences.

### Dental Education in the UK

KCLDI is one of 13 long established dental schools in the UK with two allied institutions and one new school having opened in the past two years, following an expansion in undergraduate dental student places in the UK [6]. There is a fixed quota of UK places with the majority of students entering a five-year dental degree programme following successful completion of secondary education. The percentage of overseas students (non-EU) is capped at 5%. The vast majority of the 'home' students comprise UK nationals. Intake policies vary but typically include UKCAT tests of professionalism and interviews [7].

### Career options

The pressures for change in the dental workforce have been outlined in detail in the earlier publications in this series [3,2,5], and a recent opinion paper by Gallagher and Wilson [8]. Change should enhance the range of opportunities open to dentists for professional development such as the expansion of the role of Dentists with a Special Interest within the NHS [9-14], or specialist status [15]. The balance between NHS and private dental care is changing [16], and dental students find themselves having to look closely at financial issues in light of student debt [3]. Increasing numbers of women are coming into dentistry and other health professions; this has resulted in debate over the 'feminisation' of the medical and dental professions within the UK [17-20], and North America [21], together with discussion over their leadership potential globally [22]. This is in marked contrast to Eastern Europe where there are increasing numbers of men entering what was a female dominated profession in countries such as Bulgaria [23]. Together these highlight the need for greater understanding of workforce trends and career plans and expectations to inform workforce planning.

The objective of this aspect of the study was to examine the long-term goals and perceived influences on final year dental students' professional careers.

## Methods

This study, which received research ethics approval from King's College London (KCLREC: 03/04-109) has been described by Gallagher et al., [2,3]. In essence, the population survey of fifth-year students at KCLDI was carried out immediately following finals examinations in June 2005. Of the 140 students in the year, 57% were female and 70% Asian. Dillman's (2000) approach to the conduct of a questionnaire survey was used to maximise the response rate. The questionnaire instrument covered the following five areas: vision of dentistry (why they had chosen it as a career), short-term career aspirations, long-term career aspirations, influences on their career and personal details. This questionnaire may be viewed at http://www.fgdp.org.uk/journals/pdc/articles/v15n3app1.pdf

Questions on their long-term career aspirations explored aspects of their professional lives 10 or more years post-graduation: type of role, area of interest, anticipated setting and working patterns. Factors which would influence their working patterns and career choices were also explored. Students were asked about the influences relating to systems of NHS and private dental care, including factors that would encourage them to practice NHS dentistry, the level of private care they anticipated providing and the reasons for the choice.

Questions were a mix of open questions and closed questions with factors known to influence future professional career choices which the students rated on a five-point Likert Scale from 'Strongly Agree' (Score - 1) to 'Strongly Disagree' (Score - 5).

Univariate analysis was undertaken to present an overview of the findings from this population study, together with an analysis by age, sex, and ethnicity, direct or mature entry to this course of study. Differences between groups were examined using the Chi-squared test for linear trends across the rated questions.

## Results

The results are presented in four sections: first, an overview of the respondents; second, long-term career goals and influences; third, proposed commitment to dentistry in relation to working patterns: fourth and finally, commitment to healthcare systems and the influences thereon. The influences of sex, age, ethnicity and mode of entry on long-term professional career were examined for each of the dimensions, but only reported where significant differences were identified.

### Respondents

A 90% response was achieved (n = 126) amongst 140 students in the final year of KCLDI BDS programme. Full details of respondents, who mirrored the overall profile of the year, have been published [3]. The modal age was 23 years (59%), the majority having been direct entry from school into the five-year programme or following a gap year, with a range from 22-33 years, 10.5% of whom were mature students. Females were in the majority (58%). Asians (70%) were the largest ethnic group, followed by white students (22%). Around three-quarters of Asians (77%) identified themselves as 'Indian'. Female Asians were the largest sub-group (41%; n = 52).

### Long-term career goals

Analysis of the role, setting and area of practice demonstrated that the group of respondents were fairly evenly split between three areas of future practice: 'dentist with a special interest' (27%; n = 34); 'primary dental care practitioner' (26%; n = 33); becoming a 'specialist' (25%; n = 32); and those who did not know (19%; n = 24), with only two percent (n = 3) of respondents identifying other options.

**Figure 1:**
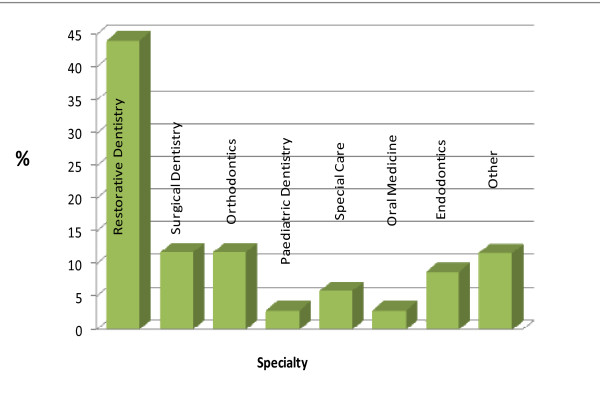
**Proposed future type of 'Dentist with a Special Interest' of KCLDI final year students (n = 34)**.

**Figure 2:**
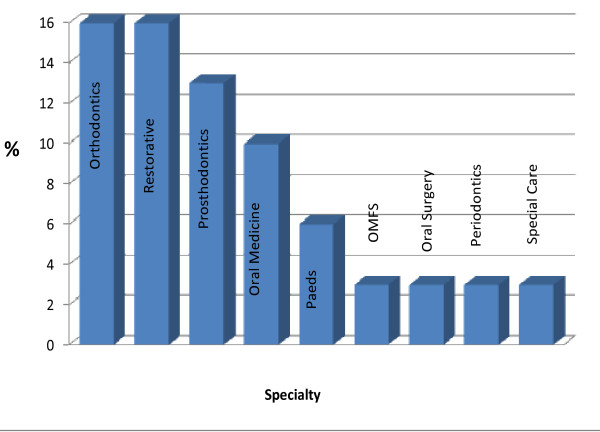
**Proposed future type of specialty of KCLDI final year students (n = 126)**.

A detailed breakdown of respondents who anticipated becoming either a 'dentist with special interest' (n = 34) or a 'specialist' (n = 32) is provided in Figures 1 and 2 respectively. Of the respondents who anticipated becoming a 'dentist with special interest', the majority (44%, n = 15) opted for the field of 'restorative dentistry' (Figure 2) and the most preferred setting for working as a 'dentist with a special interest' was 'high street practice' (70%). High street practice is the term used to denote a specialist who works in a community-based dental surgery or office rather than in a hospital.

Of those who intended to become a 'specialist', the most popular specialties were 'restorative dentistry' (16%, n = 5) and 'orthodontics' (16%, n = 5) in equal proportions (Figure 2). (Restorative dentistry in the UK is the study, diagnosis and integrated effective management of patients with diseases of the oral cavity, the teeth and supporting structures including the care of those who have additional needs associated with disability. Treatment provision involves the rehabilitation of the teeth and the oral cavity to functional psychological and aesthetic requirements of the individual patient including the co-ordination of multi-professional working to achieve these objectives. Its scope includes all the activities associated with Endodontics, Periodontics and Prosthodontics.) The preferred settings for specialists were 'hospital' (41.5%, n = 13) followed by 'high street practice' (31.5%, n = 10).

Of the 33 respondents who anticipated becoming a 'primary dental care practitioner', 52% (n = 21) wanted to be a practice owner, either joint (33%; n = 11) or sole (29%; n = 10), whereas 21% (n = 7) anticipated being an 'associate'. In relation to the nature of practice, the majority (61%; n = 20) identified that they preferred to work in 'team practice' with only 30% (n = 10) preferring to work in a 'small surgery'.

#### Area of interest by sex

**Figure 3:**
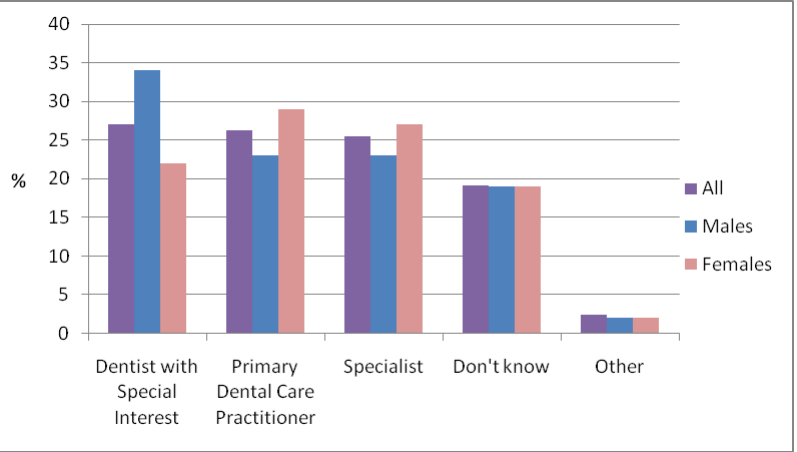
**Proposed future area of practice by sex (n = 126)**.

The majority of male respondents anticipated becoming a 'dentist with a special interest' (34%, n = 18), followed by 'primary dental care practitioner' (23%, n = 12) and 'specialist' (23%, n = 12) in equal proportions (Figure 3).

Restorative dentistry was the preferred area of interest for the majority of males (56%; n = 10). Of the 12 male respondents who anticipated pursuing a career as a 'primary dental care practitioner', just over half (58%; n = 7) expected to be 'practice owner (sole)' and work in 'team practice'. Among those who planned to follow the 'specialist' route (n = 12), three (25%) preferred 'restorative dentistry', wishing to work across a range of different settings, the most popular being 'high street practice' (42%, n = 5).

In contrast, female respondents were more likely to anticipate working as a 'primary dental care practitioner' (29%; n = 21) than other options (Figure 3). Twenty-seven percent (n = 20) planned to work as 'specialist' and 22% (n = 16) expected to pursue a career as a 'dentist with a special interest'. Of those who wished to work as a 'primary dental care practitioner', there was equal interest in being an 'associate' (33%, n = 7) and 'practice owner - joint' (33%, n = 7). The majority (62%; n = 13) anticipated working in 'team practice' followed by 'small surgery' settings (24%; n = 5). Of those who wished to practice as 'specialist' (n = 20), the main area of interest was 'orthodontics'.

### Career contribution to profession and systems

#### Full-time or part-time

Only 60% of total respondents anticipated working full-time in the long-term (Table 1). Just over one quarter (27%; n = 34) of respondents anticipated working part time. A minority did not know or did not respond to this question. Significant differences were observed between male and female responses with 52% of females considering working full-time in the long-term compared with 79% males (p = 0.00).

**Table 1:** Anticipated working patterns of KCLDI final year students by sex (n = s126)

	All		Males		Females	
Working pattern	Number	%	Number	%	Number	%
Full-time	76	*60*	40	*78*	36	*52*

Part-time *	34	*27*	8	*16*	26	*38*

Don't know	10	*8*	3	*6*	7	*10*

Not answered	6	*5*	2	*4*	4	*8*

Note: * Females > males; p = 0.0015

#### Number of sessions

**Figure 4:**
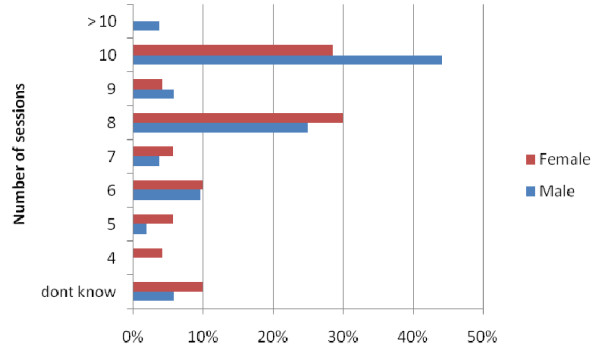
**Anticipated future workload of KCLDI final year students (n = 121)**.

When questionned regarding sessions the responses were revealing, just over one third of respondents 37% (n = 45) planned to work 10 or more sessions per week and 32% (n = 40) eight or nine sessions (Figure 4). The mode for males was 10 sessions (44%) and for females eight sessions (30%).

**Figure 5:**
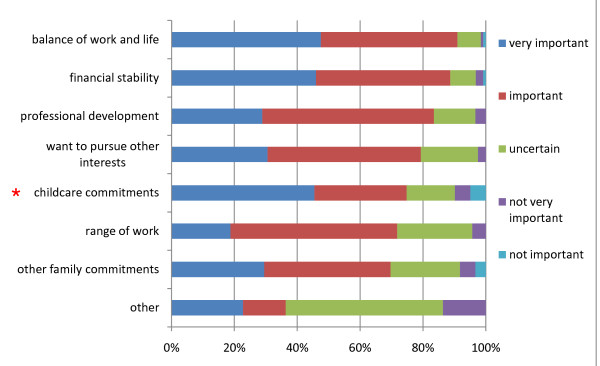
**Perceived influences on number of future sessions reported by KCLDI final year dental students (n = 123)**. Note: * Females > males; p = 0.00

In relation to the influences on the number of sessions worked, 80% or more of respondents identified 'work-life balance', 'financial stability' and 'professional development' as important or very important influences on the number of sessions worked (Figure 5). Analysis revealed only one influence showed a significant difference between males and females (Table 2); females were significantly more likely to rate childcare commitments as an important influence on their future working capacity when compared with males (p = 00).

**Table 2:** Influences on number of sessions worked: significant differences by sex (n = 126)


**Issue**	**Males**	**Females**	**Significance**

Re-training facilities after career break	68%	85%	p = 0.017

Assistance with student debt	69%	84%	p = 0.006

Child care support	56%	73%	p = 0.011

Incentives to work in deprived areas	54%	70%	p = 0.051

#### Working in the private sector

Of the 103 respondents who responded to the question exploring work in the private sector, 21% were not sure of their commitment, 40% reported that they would undertake up to, and including, half of their work privately. However, only six percent of those who responded to this question indicated that they would only work privately.

#### NHS working and influences

**Figure 6:**
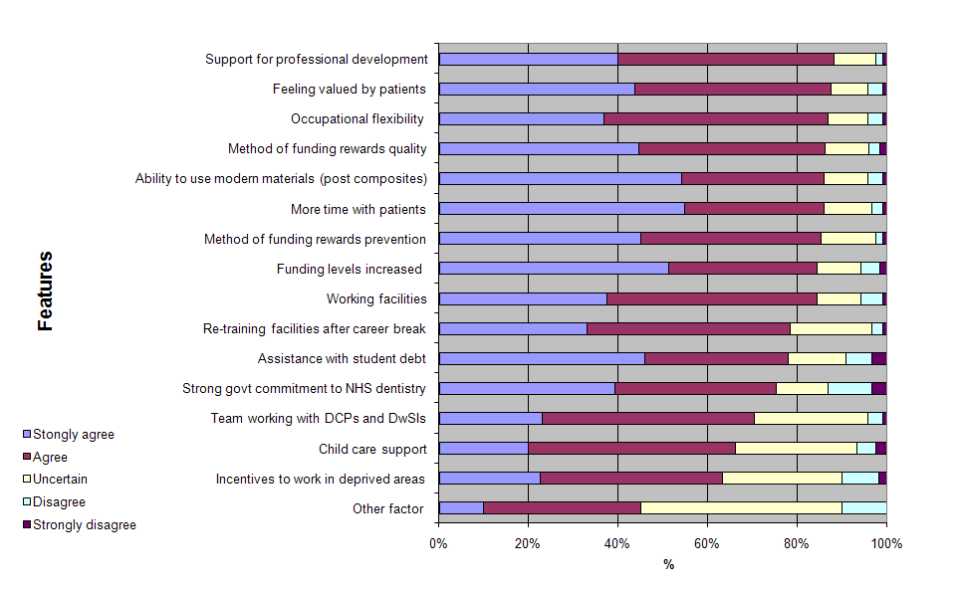
**Features perceived as enhancing the attractiveness of the NHS by KCLDI final year students (n = 126)**.

A wide range of factors were considered important or very important in making the NHS attractive, led by support for professional development (88.3%) and feeling valued by patients (87.6%) (Figure 6).

There were a number of factors on which females were significantly more likely than males to consider important regarding to working in the NHS (Table 2). These related to 'childcare support', 'retraining facilities after career break', 'assistance with student debt' and 'incentives to work in deprived areas'.

### Overall influences on long-term careers

**Figure 7:**
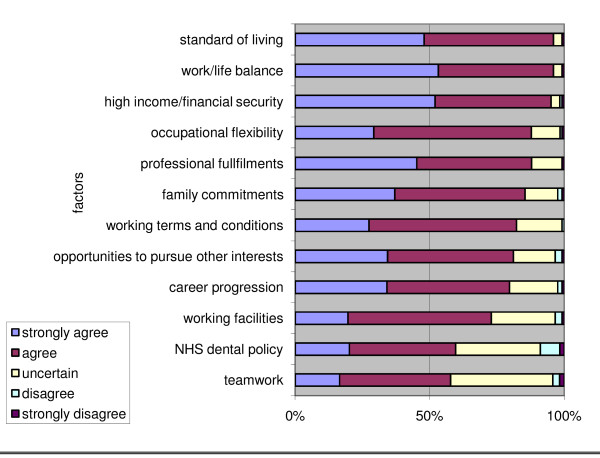
**Perceived influences on long-term career overall by KCLDI final year students (n = 126)**.

Overall influences on long-term careers receiving most support from final year students as important or very important related to personal factors such as 'standard of living', work-life balance' and 'high-income/financial security' (Figure 7). Issues such as 'NHS dental policy' and 'team working' were much less prominent, albeit that they were considered important for over half of respondents.

## Discussion

### Strengths and limitations

This research contributes to knowledge providing insight into the views of students' and recent graduates on future practice [5,24,25]. The timing of this study, just prior to introduction of the new dental contract in England, could be considered as a limitation. The survey was completed when there was a lot of uncertainty around the future direction of primary dental care and that may have influenced the findings. Furthermore, it has not yet been possible to date to follow-up these graduates to investigate possible changes in the career expectations and influences subsequent to graduation. Whilst it is recognised as a limitation that these are the views of students' from only one dental school and it is also likely that this large establishment in London may attract and retain a different type of student than other smaller dental schools, thus its undergraduates may not be representative of the national picture, it is the largest of the UK schools. Thus its students will make a significant contribution to the professional view nationally and thus the findings are of wider relevance. The lack of significance in relation to ethnicity and mature students in these findings may be related to the predominance of Asians in this student population. These points highlight the need for future research, where possible, to be conducted across institutions, as already recommended [3,5].

### Future careers

Personal factors are perceived as key influences on future careers. Part of this may be the fact that final year dental students have not begun to experience working life, let alone develop a commitment to a particular job or role. This construct does however fit with the qualitative and quantitative findings on motivation for choosing dentistry as a professional career, precisely because the job provides flexibility and enables them to have a 'contained professional career'. Interestingly, although professional careers do not come out as the most important influences in the choice of career [3] or short-term plans [5], clearly professional development is important to these students with over half claiming an interest in becoming a 'specialist' or a 'dentist with a special interest' (the latter holding qualification and skills somewhere between a generalist and specialist) [9], at this stage in their professional careers. The numbers proposing to consider working in each professional area are small and must be treated with caution; however, the findings are helpful in that they provide evidence of the range of future career plans being considered. The level of interest in becoming a 'dentist with a special interest' was notable, given that this role is competency based, does not yet have a clear professional status, and training programmes are only just being developed. Given that the majority of dentists have historically worked in primary care, the correspondingly low level of interest in remaining a primary dental care practitioner in the long-term is also noteworthy as it has implications for primary care capacity. Furthermore, this school has academically bright students, many of whom may have higher grades than medical counterparts; hence there is the potential for boredom amongst a group of high achievers who respond to new challenges and opportunities. However, similar findings have been reported in the recent study of Welsh VDPs [25], which may represent a generational shift in perspective, possibly facilitated by changes in college diplomas for postgraduate development [26] and the requirement for fewer essential qualifications for entry to specialist training [27].

### Implications of findings

An important issue to consider is whether career aspirations will contribute to meeting population oral health needs in future? Given patterns of oral health and our ageing population, much dental care is increasingly routine and the majority of complex needs will be in middle aged and older people who have clear expectations of dental professionals and NHS dental care [28,29]. They are increasingly likely to seek, and require, complex restorative care; however, as they progress through old-age and become increasingly vulnerable, simpler clinical measures may be more appropriate, but their medical management may be more complex [30]. The findings of this study suggest that many new graduates support the concept of team-working and working in larger practices. This view sits well with a model of future working proposed by Gallagher and Wilson [8], whereby dentists are leaders of larger dental teams, use their high level skills in undertaking complex techniques/procedures, and harness the support of auxiliary staff in providing routine and preventative care.

The desire for postgraduate and specialist development raises the need to review the distribution of all the funding which goes into dental education and training. Traditionally the NHS has borne these costs; however, with the component specialties of Restorative Dentistry, as practiced in the UK - Prosthodontics, Endodontics and Periodontics - dentists have had to self-fund, therefore they cannot be expected to contribute to the NHS as they will have to pay off their fees and the opportunity costs of studying. Also, within the new NHS dental contract in England there is evidence of much less complex care being provided [31,32]. Oral and dental care must play an important role in the health and wellbeing of the population and therefore cannot be relegated to the private sector, but planned for, funded and provided within the NHS, as with the rest of healthcare.

### The gender debate

Given the findings of this research in relation to anticipated roles and working patterns, it is not possible to ignore the long-term views on professional careers, of males and females. We can not ignore the fact that the proposed contribution of females to the workforce differs and their long-term contribution may be more limited in volume and nature, with anticipated family childcare issues influencing their views. These findings are supported by recent research amongst Welsh VDPs [25], where females were suggesting that they were more likely to work part-time. Interestingly, the findings have parallels in issues identified by the Royal College of Physicians of England in 2004, where there have been concerns that women in medicine are more likely to look for part-time and job-share work in primary care and they are less likely to be represented in the upper echelons of the profession [20]; they go on to suggest that the system does not operate in a way that sustains these choices and the need to 'reorganise radically the way things are done in medicine, so that women can find congenial careers across the whole range of medical specialties'. But do they want to? Emerging dentists rarely have the pressure of children and yet held these views. In fact going back to the first stage of this project, there was evidence that dentistry had been chosen as a 'professionally contained career' [2,2,33], and specific references amongst females that they saw it as providing the flexibility to combine professional working life with parenthood in later life [3,34]. However, the emphasis on females ignores the fact that only 80% of males anticipated working full-time, a finding which has wider support in the literature [18,25], with recognised implications for future workforce planning [8]. Further research would be helpful to examine the views of Asian females in greater detail, across institutions and longitudinally, given their position in the dental workforce.

### NHS - not written off

In considering their future involvement in health care systems, clearly the students were prepared to work in the NHS, expressing similar perspectives to recent graduates [25,24]. Should their views persist, there is potential for a mixed practice economy to continue, as currently. However, as with the views of VDPs, the findings suggest that their support is dependent upon certain changes within the system to meet their values and expectations [24]. The Departments of Health and NHS organisations must take note of the factors which will draw young dentists into the system, setting the tone and value of NHS dentistry and ensuring that there is some occupational flexibility in the system. Otherwise we run the risk of losing this important resource and the opportunity to harness this potential for new ways of working in the NHS. It is also the responsibility of government to create a new system which rewards quality, prevention and enables sufficient time with patients to facilitate a professionally fulfilling career, whilst supporting team-working in appropriate premises. These issues received great support in Lord Darzi's (Minister for Health's) recent reviews of healthcare in general [35,36]. These are challenges that have been presented to the national Health Select Committee review in 2008 [37], and must be considered in the review of the new dental contract currently being established in England [32]. Although government and the NHS must set the framework, and commissioning organisations should develop more robust methods to address oral health needs, it is principal dentists and corporate bodies who will be key to transforming the future. Much depends on their ability to be effective trainers, mentors, and negotiators, considering the long-term needs of the population and future of the profession. They can, and should, be supporting professional development, quality care and prevention whilst developing larger practices [25].

## Conclusion

Long-term career plans of new graduates from this London dental school commonly embrace opportunities for professional development as well as personal issues such as work/life balance and financial income. Significant differences were identified between males' and females' long-term plans and influences. These findings have implications for health policy and workforce planning, and highlight the need to consider the views and aspirations of future members of the profession.

## Competing interests

Two of the authors (JEG and NHFW) are academic staff at King's College Dental Institute. NHFW is Dental Dean and Head of King's College London Dental Institute.

## Authors' contributions

JEG and NHFW conceived and designed the overall research programme. JG led the development of the protocol, gained ethics committee approval, oversaw the fieldwork including development of the questionnaire, contributed to the data analysis, interpretation of results and led on writing of the paper. RP conducted the fieldwork, entered the data to SPSS and contributed to the descriptive analysis as part of her Masters programme. All authors reviewed the final manuscript.

## Pre-publication history

The pre-publication history for this paper can be accessed here:

http://www.biomedcentral.com/1472-6831/9/35/prepub
